# Thiophene Derivatives as Versatile Precursors for (Hetero)Arene and Natural Product Synthesis

**DOI:** 10.1002/anie.202516780

**Published:** 2025-12-09

**Authors:** Anna Keimer, Franz‐Lucas Haut

**Affiliations:** ^1^ Freie Universität Berlin Institute of Chemistry and Biochemistry Takustr. 3 14195 Berlin Germany

**Keywords:** Heterocycles, Medicinal chemistry, Natural products, Pericyclic reactions, Total synthesis

## Abstract

Thiophenes and their saturated analogues are versatile C_4_‐building blocks used to construct structurally intricate ring systems found in various biologically active structures. For instance, thiophenes can undergo dearomative cycloaddition reactions under photocatalytic conditions forming highly substituted benzenes. Thiophene *S*‐oxides and *S*,*S*‐dioxides have emerged as valuable precursors for the construction of complex frameworks in (4 + 2)/retro‐(4 + 1) cascade reactions through cheletropic release of SO or SO_2_, respectively. In addition, 2,5‐dihydrothiophenes can be rapidly converted into furans or pyrroles via pericyclic cascade reactions. The installation of a tetrahydrothiophene followed by reductive deletion of the sulfur atom has been demonstrated to be a powerful method for the *cis*‐dialkylation of electron‐poor olefins. The ability to generate highly complex, aromatic scaffolds and quaternary stereocenters makes thiophene compounds valuable intermediates for synthesizing natural products and bioactive molecules, which are important for crop science and medicinal chemistry. This minireview provides an overview of strategies for using these sulfur‐containing heterocyclic precursors in challenging synthetic applications.

## Graphical abstract

**Hidden but Powerful**: Thiophenes and their saturated analogues have been demonstrated to be versatile C_4_‐building blocks that can be rapidly applied to natural product synthesis and the construction of functionalized (hetero)arenes. This minireview aims to provide a concise overview of the strategies and implementations of these sulfur‐containing heterocyclic precursors in challenging synthetic applications.

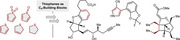

## 1 Introduction

**Figure 1:**
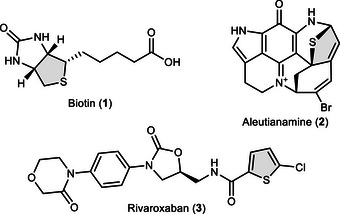
Biologically relevant molecules featuring tetrahydrothiophene (**1**), 2,5‐dihydrothiophene (**2**) or thiophene scaffolds (**3**).

The development of new synthetic methods that reveal powerful but previously unknown retrosynthetic disconnections is fundamental to facilitate all steps in drug discovery and crop science campaigns.^[^ ^1^, ^2^ ^]^ In this context, precise engineering of heterocycles via deletion, addition, or swap of individual atoms in its core structure has evolved into an elegant synthetic technology for the late‐stage diversification of advanced intermediates. Whereas these strategies could facilitate enhancing pharmaceutically important properties of biologically active structures, concurrent methods have been mainly focused on azaarenes as targets.^[^ ^3^, ^4^, ^5^ ^]^ An important class of heterocycles is based on thiophenes and their saturated congeners. These are ubiquitous in biologically active compounds such as the natural products biotin (**1**)^[^ ^6^ ^]^ and aleutianamine (**2**, Figure 1).^[^ ^7^ ^]^ Furthermore, they are considered as privileged pharmacophores in medicinal chemistry. For example, the anticancer blockbuster drug rivaroxaban features a thiophene in its core structure (**3**).^[^ ^8^, ^9^ ^]^ Due to their frequent occurrence in medicinal chemistry, crop science, and materials, thiophene derivatives are a highly desirable scaffold for establishing synthetic practices through site‐selective modification of the *S*‐heterocycle.

**Scheme 1:**
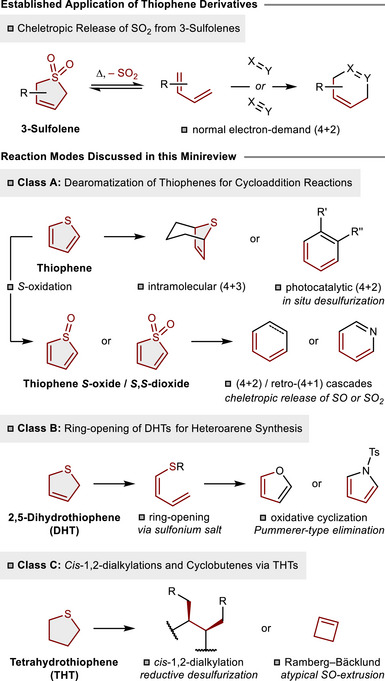
Traditional application of 3‐sulfolenes as butadiene surrogates and versatile reactivity of related thiophene derivatives (classes A–C, scope of this minireview).

Despite the paucity of studies examining the late‐stage diversification of thiophenes, there has been a notable development of related *S*‐heterocycles as powerful precursors in pericyclic transformations facilitating heteroarene synthesis and the construction of natural products. Among the thiophene derivatives, 3‐sulfolenes (2,5‐dihydrothiophene *S*,*S*‐dioxides) have emerged as the most prominent representatives. These heterocyclic sulfones have garnered significant recognition as butadiene surrogates, a distinction that arises from their characteristic cheletropic release of SO_2_ (Scheme 1, *top*).

Consequently, their synthetic utility has been demonstrated through the extensive application in Diels–Alder cycloadditions in the context of natural product synthesis. As SO_2_‐extrusion from 3‐sulfolenes has been frequently applied over the last few decades and has therefore been comprehensively reviewed,^[^ ^10^, ^11^ ^]^ this minireview aims to focus on contemporary developments in the field employing thiophenes, 2,5‐dihydrothiophenes (DHTs), and tetrahydrothiophenes (THTs) as valuable precursors for organic synthesis (Scheme 1, *bottom*). The application of thiophenes as C_4_‐building blocks has been conventionally limited due to their intrinsic aromaticity, but recent studies have demonstrated their potential as 1,3‐dienes under mild conditions, facilitated by photocatalysis or intramolecular reaction pathways (class A). A complementary approach involves the dearomative oxidation of sulfur, which accesses thiophene *S*‐oxides and *S*,*S*‐dioxides as highly reactive species for Diels–Alder reactions. Notably, this approach facilitates the effective construction of polysubstituted benzenes, pyridines or cyclohexa‐1,3‐dienes depending on the dienophile employed in the transformation. The ability to undergo (4 + 2)‐cycloadditions with electron‐rich alkenes or nitriles accompanied by the cheletropic release of SO or SO_2_ [retro‐(4 + 1)], respectively, stands in sharp contrast to the classical reactivity of 3‐sulfolene precursors, which typically react with electron‐deficient dienophiles. In addition, DHTs have been shown to serve as effective precursors for the synthesis of pyrroles and furans (class B). The *S*‐heterocycle can be readily ring‐opened through a sulfonium intermediate, and the resulting thioether can function as a handle to initiate oxidative cyclization cascades terminated by a Pummerer‐type elimination. The third section (class C) will emphasize the construction of THTs en route to cyclobutenes and vicinal *cis*‐oriented alkyl residues. Herein, a non‐classical Ramberg–Bäcklund reaction creates the four‐membered carbocycle via extrusion of SO. Moreover, otherwise challenging 1,2‐*cis*‐dialkylations can be achieved through reductive desulfurization of advanced THT derivatives.

## 2 Thiophenes

**Scheme 2:**
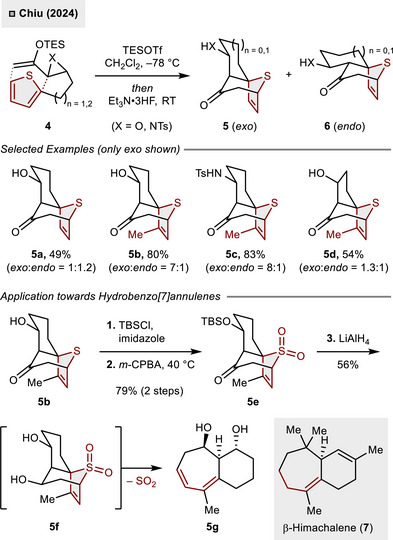
Dearomative intramolecular (4 + 3)‐cycloadditions of thiophenes toward hydrobenzo[7]annulene skeletons.

Furans have been identified as electron‐rich 1,3‐diene moieties in cycloaddition reactions, as well as useful precursors for butenolide or γ‐lactone synthesis via oxidation or hydrolysis, respectively.^[^ ^12^ ^]^ However, the heavier chalcogen analog, thiophene, has scarcely been explored in these applications. This may be due to its higher aromaticity compared to furans, which makes thiophenes significantly less reactive in dearomative transformations.^[^ ^13^ ^]^ In preliminary work, thermal or high‐pressure conditions have forced thiophenes to successfully participate in Diels–Alder reactions. However, the synthetic scope remained limited due to the requirement of highly activated dienophiles, diminished functional group tolerance and specialized equipment needed to conduct reactions at high pressure.^[^ ^14^, ^15^ ^]^ Very recently, thiophenes were shown to participate in intramolecular, dearomative (4 + 3)‐cycloadditions with epoxy silyl enol ethers (X  =  O) or their *N*‐tosyl aziridine analogues **4** (X  =  NTs) far below room temperature (–78 °C) as reported by Chiu and coworkers (Scheme 2).^[^ ^16^ ^]^ The application of triethylsilyl triflate (TESOTf) as a Lewis acid permits smooth dearomatization, resulting in the formation of the tricyclic *exo*‐product **5** and the *endo*‐product **6**. Monosubstituted thiophenes showed almost no selectivity (**5a**, *dr* = 1:1.2), but the formation of the *exo*‐product improved drastically when using 3‐alkyl substituted thiophenes (**5b** and **5c**). However, a shorter epoxide chain linker (n  =  1) decreased selectivity as only minor *exo*‐selectivity was observed in **5d** (54%, *dr*  = 1.3:1). Ultimately, the tricyclic compound **5b** was converted into sulfolene **5e** via alcohol protection (TBSCl, imidazole) and *S*‐oxidation (*m*‐CPBA). Although the cheletropic release of SO_2_ was unsuccessful under thermal conditions, SO_2_‐extrusion could be realized employing LiAlH_4_ as both a reductant and an SO_2_‐scavenger. Initial desilylation and reduction led to the 1,3‐diol **5f**, which favored the pericyclic extrusion of SO_2_ due to its chair conformation. The obtained bicycle **5g** is analogous to the core of β‐himachalene (**7**), a skeleton that is inaccessible when employing acyclic dienes instead of thiophenes due to the prevalence of (3 + 2)‐cycloaddition pathways.

**Scheme 3:**
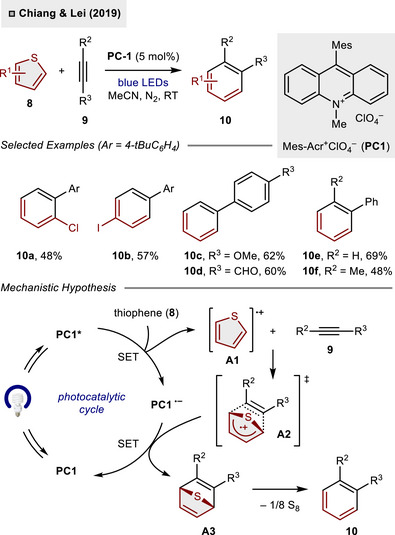
Photocatalytic dearomative (4 + 2)‐cycloaddition of thiophenes with non‐activated alkynes.

The advent of visible‐light photocatalysis and the development of strongly oxidizing organophotocatalysts, such as 9‐mesityl‐10‐methylacridinium perchlorate (**PC1**), have established reaction conditions conducive to the activation of thiophenes at ambient temperature and pressure. In 2019, the photocatalytic (4 + 2)‐cycloaddition of various thiophene derivatives **8** with alkynes **9** was established by Chiang and Lei to access a range of polysubstituted benzenes **10** employing **PC1** (5 mol%) under blue light irradiation (Scheme 3).^[^ ^17^ ^]^ In contrast to previous, harsh reaction conditions, functional groups such as halogens (**10a**, **10b**) or aldehydes (**10d**) are well tolerated under the photochemical reaction conditions. Furthermore, internal alkynes can successfully participate in the reaction, enabling the synthesis of *ortho*‐disubstituted benzene **10f** in albeit moderate yield (48%). From a mechanistic perspective, the initial photooxidation of the thiophene from the photoexcited state PC1* is postulated to result in the formation of the cationic open‐shell intermediate **A1**. Subsequent (4 + 2)‐cycloaddition with alkyne **9** accesses first bicycle **A2** and then **A3** through single electron transfer (SET). This process enables the recovery of **PC1** from **PC1^•–^**. The final release of sulfur (S_8_) unveils the benzene product **10**. The concept of photooxidative dearomatization has been further developed in ring‐expansion reactions with bicyclo[1.1.0]butanes to produce unusual eight‐membered ring motifs and rearranged tricycles through the insertion of cyclobutanes in the thiophene core.^[^ ^18^, ^19^ ^]^ As demonstrated in recent studies, boryl radicals have been found to effectively trigger dearomative boron insertion into thiophenes through one‐electron processes.^[^ ^20^ ^]^

**Scheme 4:**
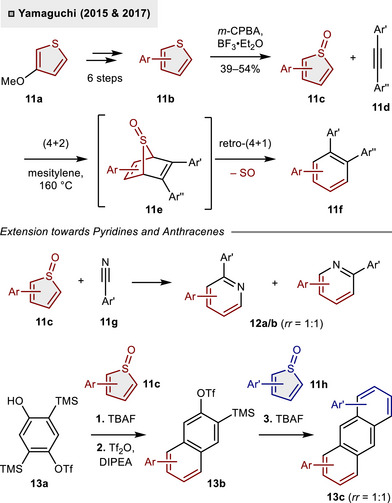
Programmable synthesis of polyarylated (hetero)aromatics utilizing thiophene *S*‐oxides.

Another strategy to break the aromaticity of thiophenes is based on *S*‐oxidation to generate the corresponding sulfones. The non‐aromatic thiophene *S*‐oxides and *S*,*S*‐dioxides are highly reactive intermediates and, consequently, rapidly engage in cycloaddition reactions. In 2015, Yamaguchi and coworkers reported the programmable synthesis of fully‐arylated benzenes employing thiophene *S*‐oxides as 1,3‐diene equivalents (Scheme 4).^[^ ^21^ ^]^ The *S*‐heterocyclic precursor could be modularly diversified via consecutive Pd‐catalyzed cross‐couplings starting from commercially available 3‐methoxythiophene (**11a**). Following a series of six steps, the desired thiophene **11b** was obtained. This compound contains four different aromatic residues and was subsequently oxidized with *m*‐CBPA resulting in the formation of *S*‐oxide **11c** (up to 54% yield). At elevated temperatures (160 °C) in mesitylene as the solvent, **11c** underwent a cascade reaction with alkyne **11d** based on initial (4 + 2)‐cycloaddition (**11e**) followed by retro‐(4 + 1)‐cycloaddition via cheletropic extrusion of SO. In addition to the synthesis of hexaarylated benzenes **11f**, the developed protocol was found to be applicable for the synthesis of pyridines **12a/b** (*rr *= 1:1) when employing aryl nitriles **11g** as dienophiles. The strategy could be further extended to prepare octaaryl anthracenes.^[^ ^22^ ^]^ For this purpose, a twofold cycloaddition of two different thiophene *S*‐oxides (**11c**, **11h**) has been developed which relied on Kobayashi‐type precursor **13a**. Exposure to **11c** and tetra‐*n*‐butylammonium fluoride (TBAF) resulted in the targeted cyclization cascade, while the second trimethylsilyl (TMS) group of **13a** remained unaltered. Triflation then accessed naphthalene **13b**, thereby enabling a subsequent (4 + 2)/retro‐(4 + 1) sequence through the generation of a transient naphthalyne species. This ultimately enabled the incorporation of a second *S*‐oxide **11h**, thereby facilitating access to the polyfunctionalized anthracene derivative **13c** (*rr *= 1:1).

**Scheme 5:**
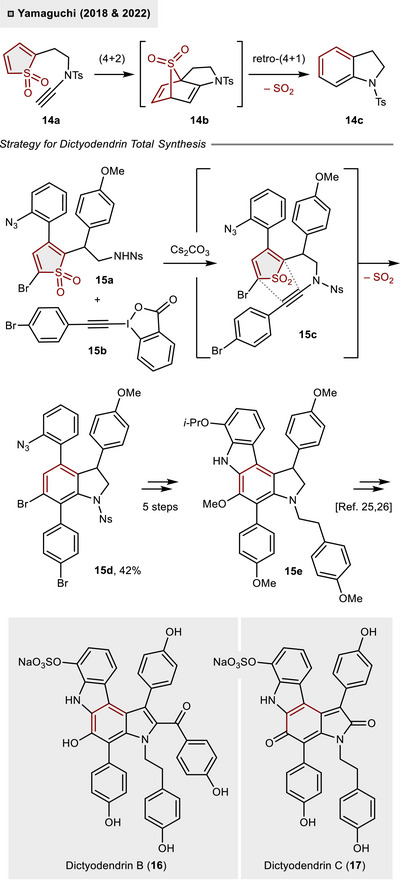
Indoline construction via intramolecular (4 + 2)‐cycloaddition of thiophene *S*,*S*‑dioxide and ynamide.

Later, the group employed a related synthetic strategy in the construction of the central indoline core of dictyodendrin natural products (Scheme 5).^[^ ^23^, ^24^ ^]^ In contrast to the preceding studies, the arene motif was formed through an intramolecular (4 + 2)‐cycloaddition of a thiophene *S*,*S*‑dioxide with an ynamide (**14a**→**14c**). The cyclization precursor **15c** was generated in situ through the *N*‐alkynylation of 4‐nitrobenzene‐sulfonamide (Ns‐amide) **15a** with hypervalent iodine reagent **15b** under basic reaction conditions (Cs_2_CO_3_). Upon formation of the crucial C(sp)–N bond, **15c** engaged in a cycloaddition/SO_2_‐extrusion cascade, thereby unveiling indoline **15d**. A total of five additional steps were required to prepare **15e**, which has been identified as a pivotal intermediate in the synthesis of dictyodendrin B (**16**) and dictyodendrin C (**17**).^[^ ^25^, ^26^ ^]^

**Scheme 6:**
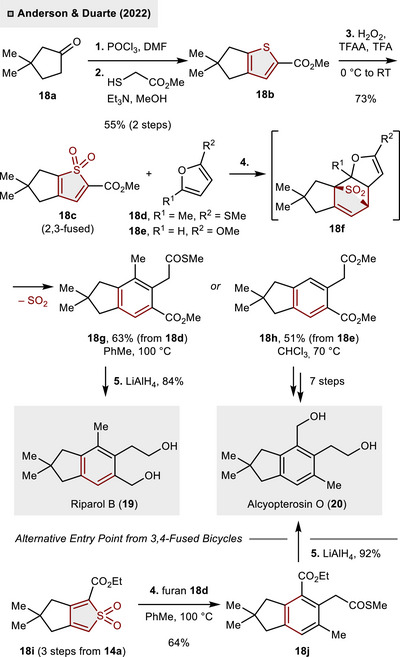
Total synthesis of illudalane sesquiterpenes through (4 + 2)‐cycloadditions of thiophene *S*,*S*‑dioxides.

In 2022, the efficacy of fused bicyclic thiophene *S*,*S*‐dioxides as synthetic intermediates in the construction of the highly substituted benzene core of illudalane sesquiterpenoids was demonstrated by Anderson and Duarte.^[^ ^27^ ^]^ As illustrated in Scheme 6, the conversion of 3,3‐dimethyl‐cyclopentanone (**18a**) into thiophene **18b** occurred in two steps (55% yield). Subsequent oxidation employing in situ generated trifluoroperoxyacetic acid (from H_2_O_2_, TFAA and TFA) led to the formation of sulfone **18c** in 73% yield. When exposed to polysubstituted furans **18d** or **18e** as dienophile, a rapid (4 + 2)‐cycloaddition took place resulting in the formation of benzenes **18g** or **18h,** respectively, in up to 63% yield. Presumably, the reaction proceeded via intermediate **18f** followed by SO_2_‐extrusion under thermal reaction conditions (70 – 100 °C). From **18g**, LiAlH_4_‐mediated reduction of both, thioester and ester moieties, led to the direct synthesis of riparol B (**19,** 84%). On the other hand, seven additional steps were required to synthesize the natural product alcyopterosin O (**20**) from **18h**. Alternatively, **20** could be accessed from 3,4‐fused thiophene **18i** in a more step‐efficient manner. Once more, the (4 + 2)‐cycloaddition and cheletropic release of SO_2_ accessed benzene derivative **18j** (64%), which could be directly converted into alcyopterosin O (**20**) through complete reduction of the ester functions (LiAlH_4_, 92%). The efficient access to polysubstituted indane scaffolds through intermolecular (4 + 2)‐cycloadditions of thiophene *S*,*S*‐dioxides and furans cumulated in the successful preparation of eight additional illudalane sesquiterpenoids.

**Scheme 7:**
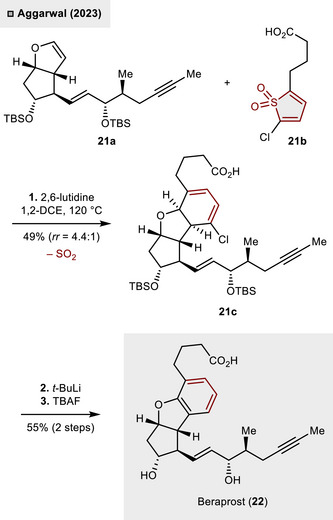
Total synthesis of beraprost (**22**) through (4 + 2)_‐_cycloaddition followed by dehalogenation–aromatization.

One year later, thiophene *S*,*S*‑dioxides were successfully applied by Aggarwal and coworkers in the total synthesis of the antiplatelet pharmaceutical agent, beraprost (**22**).^[^ ^28^ ^]^ Consistent with prior methodologies, the *S*‐heterocycle was utilized as the C_4_‐fragment to construct an annulated benzene motif, as illustrated in Scheme 7. Herein, 2,3‐dihydrofuran **21a** underwent the anticipated (4 + 2)‐cycloaddition with sulfone **21b**. The spontaneous SO_2_‐release led to the formation of cyclohexa‐1,3‐diene derivative **21c** in moderate regioselectivity and yield (*rr*  =  4.4:1, 49%). While the aromatization process proved to be challenging, treatment with *t*‐BuLi eventually resulted in the initiation of a dehalogenation‐aromatization sequence. Final deprotection of the silyl ethers revealed beraprost (**22**), with a yield of 55% achieved over the course of two steps.

**Scheme 8:**
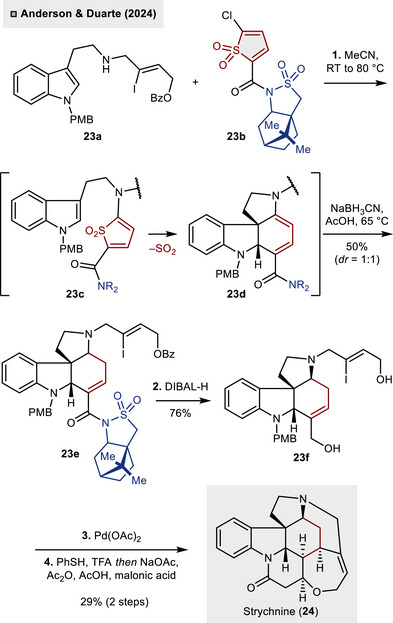
Auxiliary‐controlled (4 + 2)_‐_cycloaddition of thiophene *S*,*S*‑dioxides for the enantioselective assembly of *strychnos* alkaloids.

Recently, the utilization of thiophene *S*,*S*‑dioxides has been disclosed by Anderson and Duarte in the context of the collective synthesis of *strychnos* alkaloids.^[^ ^29^ ^]^ In contrast to previous studies, the thiophene functions as an entry point to a highly substituted cyclohexene derivative. As illustrated in Scheme 8, the incorporation of a camphor‐derived amide at the thiophene backbone **23b** promoted an auxiliary‐controlled (4 + 2)‐cycloaddition with indole **23a**, which could be obtained in a single step from PMB‐protected tryptamine. The anticipated reaction cascade involves an initial halide substitution (**23c**), followed by an intramolecular (4 + 2)‐cycloaddition, culminating in the cheletropic release of SO_2_. Unfortunately, the reduction of the intermediate dienamine **23d** (AcOH, NaBH_3_CN) yielded the tetracycle **23e** as a 1:1 mixture of diastereomers (50%). The diastereoselectivity of the process could be significantly enhanced by implementing an intermolecular (4 + 2)‐cycloaddition approach, albeit at the expense of an additional synthetic step. Treatment of **23e** with DIBAL‐H resulted in the concurrent cleavage of the auxiliary and benzoyl protecting groups, thereby facilitating the formation of diol **23d**. The synthesis of strychnine (**24**) was then completed through a two‐step procedure: Initially, Heck reaction using Pd(OAc)_2_ was employed to construct the intending piperidine ring, thereby generating the Wieland–Gumlich aldehyde (not depicted). Second, the PMB‐deprotection (PhSH, TFA) was followed by the formation of the lactam and tetrahydro oxepine rings, which facilitated access to the natural product **24**. In addition to strychnine, seven distinct *strychnos* alkaloids could be synthesized in an enantioselective manner through the implementation of the auxiliary‐controlled (4 + 2)‐cycloaddition strategy.

**Scheme 9:**
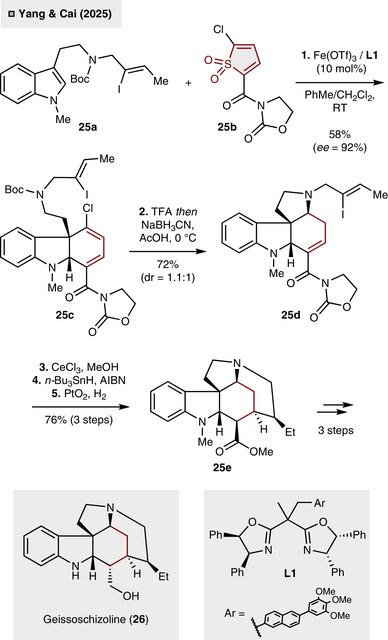
Fe‐catalyzed, enantioselective synthesis of geissoschizoline (**26**) from a thiophene *S*,*S*‑dioxide and indole.

Concurrently, Yang and Cai reported the feasibility of Fe‐catalysis to be applied for the construction of enantioenriched 1,3‐cyclohexa‐1,3‐dienes from indoles and thiophene *S*,*S*‑dioxides (Scheme 9).^[^ ^30^ ^]^ The *N*‐acyloxazolidinone group installed in the C_2_‐position of **25b** has been identified as being essential for stereocontrol, as it provides a suitable coordination site for the Fe‐catalyst. The reaction utilizes Fe(OTf)_3_, BOX‐ligand **L1** and can be performed at room temperature (25 °C) to yield the cycloadducts in generally high yield and enantioselectivity. This strategy was subsequently employed in the total synthesis of alkaloid geissoschizoline (**26**). Initially, indole **25a** underwent smooth cycloaddition with thiophene derivative **25b** to access tricycle **25c** in 58% yield (92% ee). Subsequent to Boc‐deprotection (TFA), pyrrolidine **25d** was created upon exposure to reductive reaction conditions (72%, *dr*  = 1.1:1). In the next three steps, oxazolidine cleavage (CeCl_3_, MeOH), radical‐mediated cyclization (*n*‐Bu_3_SnH, AIBN) and hydrogenation employing PtO_2_ were exploited to construct the pentacyclic core of the natural product **25e** in 76% yield over three steps. To that end, three additional steps were required to complete the total synthesis. These steps include the deprotection of the *N*‐Me indoline and inversion of the configuration of the ester function. Notably, the present study as well as the auxiliary‐based approach by Duarte and Anderson serve as seminal examples in the field of synthesizing cycloadducts under a high degree of enantioselectivity through the delineated (4 + 2)‐cycloaddition/ (4 + 1)‐cycloreversion strategy.

Beyond their powerful application as C_4_‐building blocks for challenging carbocycle formation in total synthesis, 2,3,4,5‐tetrabromothiophene *S*,*S*‐dioxide (SOgen) has found valuable applications as SO_2_‐surrogate, for instance, in the context of Pd‐catalyzed aminosulfonylation reactions.^[^ ^31^ ^]^

## 3 Dihydrothiophenes

**Scheme 10:**
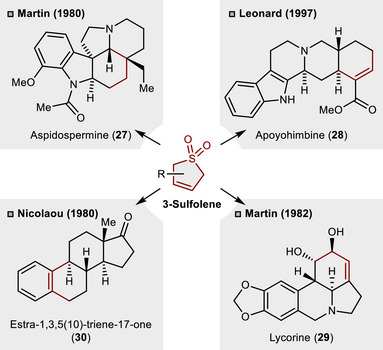
Selected applications of 3‐sulfolenes as versatile butadiene surrogates in total synthesis.

3‐Sulfolenes are well recognized as butadiene surrogates through the cheletropic release of SO_2_ and have been extensively applied in Diels–Alder cycloadditions in the context of natural product synthesis.^[^ ^10^, ^11^ ^]^ As depicted in Scheme 10, these intermediates have functioned as synthetically versatile C_4_‐building blocks in the total synthesis of the alkaloids aspidospermine (**27**, Martin),^[^ ^32^ ^]^ apoyohimbine (**28**, Leonard)^[^ ^33^ ^]^ and lycorine (**29**, Martin).^[^ ^34^ ^]^ In addition, a 3‐sulfolene intermediate has been utilized to prepare the steroid estra‐1,3,5(10)‐triene‐17‐one (**30**, Nicolaou).^[^ ^35^ ^]^

**Scheme 11:**
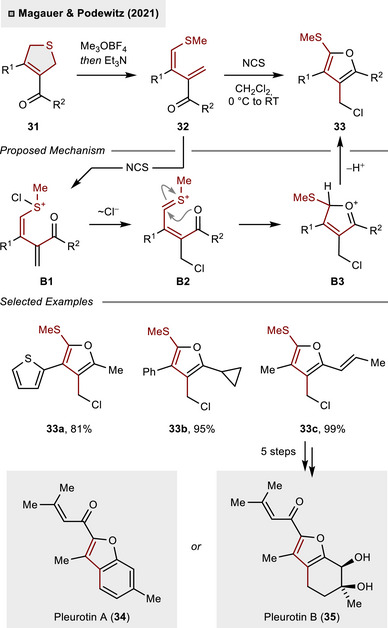
Skeletal rearrangement of DHTs to furans and application to bisabolene total synthesis.

In contrast to the cheletropic SO_2_‐extrusion, 3‐sulfolenes have been shown to participate in Pd‐catalyzed cross‐couplings. This process has been demonstrated to be a useful method for the stereoselective synthesis of arylated 1,3‐dienes through base‐promoted C–S bond functionalization.^[^ ^36^, ^37^ ^]^ In 2024, the ring‐opening under basic conditions was also employed to reorganize the skeleton of DHT sulfoximines to chiral cyclic sulfinamides by means of organocatalysis.^[^ ^38^ ^]^ However, the corresponding DHTs have only recently been investigated as C_4_‐building blocks serving as suitable synthetic precursors to construct tetrasubstituted furans, as demonstrated by Magauer and Podewitz (Scheme 11).^[^ ^39^ ^]^ Herein, the *S*‐heterocycle **31** was efficiently converted into 1,3‐diene **32** through initial methylation and base‐promoted ring‐opening. It has been demonstrated that exposure to *N*‐chlorosuccinimide (NCS) resulted in a Pummerer‐type cyclization, which served as a key step in the assembly of structurally complex furans **33**. Preliminary experimental and computational studies suggest that the reaction may proceed via a Pummerer‐type mechanism (**B1**→**B3**). Upon initial *S*‐chlorination of **32** with NCS, substoichiometric amounts of HCl trigger C(sp^3^)–Cl bond formation to access thionium species **B2**, which rapidly undergoes ring‐closure and final deprotonation to **32**. The method demonstrates a high degree of tolerance for functional groups including heteroarenes (**33a**), cyclopropanes (**33b**) and olefins (**33c**). The latter could effectively be applied to the total synthesis of bisabolene natural products, providing access to pleurotin A (**34**) and pleurotin B (**35**) in five steps after furan formation.

**Scheme 12:**
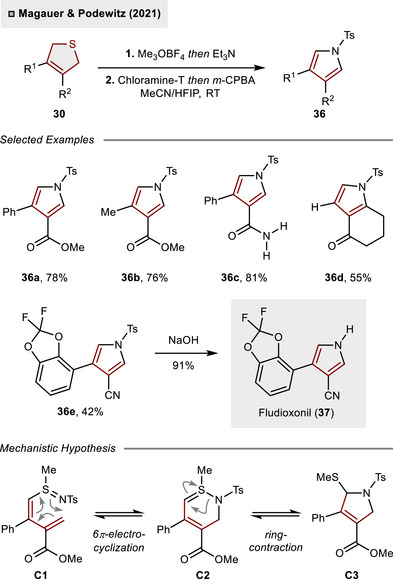
Synthesis of pyrroles from DHTs through a pericyclic cascade reaction.

The complementary reactivity of 1,3‐diene **32** was established through the simple variation of the oxidant to chloramine‐T. This change led to the facile formation of pyrrole **36** from DHT **30**, as illustrated in Scheme 12.^[^ ^40^ ^]^ The protocol was proven to tolerate various electron‐withdrawing groups such as esters (**36a, 36b**), amides (**36c**), ketones (**36d**) or nitriles (**36e**). Eventually, the protocol could be applied to the short synthesis of fungicide fludioxonil (**37**). Mechanistically, the transformation is likely initiated through *S*‐imidation of 1,3‐diene **31** leading to sulfilimine **C1**. Subsequent 6π‐electrocyclization accesses intermediate **C2**, which rapidly undergoes ring contraction leading to 2,5‐dihydropyrrole **C3**. In the presence of an excess of chloramine‐T, the elimination of methyl sulfide is triggered providing access to **36**.

## 4 Tetrahydrothiophenes

Thiophenes and DHTs have been extensively employed in pericyclic transformations. In contrast, their saturated congeners, THTs, have found complementary applications in the synthesis of complex molecules. In a manner analogous to the ring‐opening of DHTs, recent studies have demonstrated that photocatalytic reaction conditions under continuous flow permit the divergent ring‐expansion of THT vinyl sulfonium salts, ultimately resulting in the formation of six‐ and seven‐membered *S*‐heterocyclic products.^[^ ^41^ ^]^

**Scheme 13:**
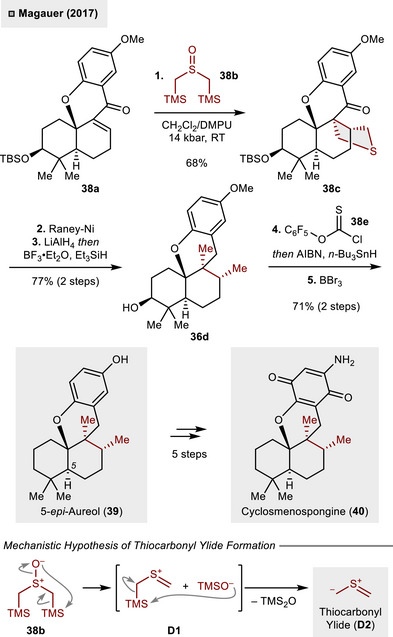
Vicinal *cis*‐dimethylation via 1,3‐dipolar cycloaddition–desulfurization strategy.

Prominently, desulfurization of THT derivatives has been identified as a powerful strategy to install vicinal *cis*‐oriented alkyl residues present in various natural products. In preliminary work toward the total synthesis of cyclosmenospongine (**40**) reported by Magauer and coworkers, the *S*‐heterocycle could be constructed through a 1,3‐dipolar cycloaddition of a putative thiocarbonyl ylide intermediate **D2** generated from sulfoxide **38b** (Scheme 13).^[^ ^42^ ^]^ From a mechanistic perspective, the 1,3‐dipolar species is hypothesized to be formed from **38b** via a pathway that is related to the sila‐Pummerer rearrangement.^[^ ^43^ ^]^ It has been established that high‐pressure reaction conditions (14 kbar) were imperative for the (3 + 2)‐cycloaddition with α,β‐unsaturated ketone **38a**, thereby producing THT derivative **38c** in 68% yield as a single diastereomer.^[^ ^44^ ^]^ The vicinal methyl groups were then unleashed through Raney‐Ni‐mediated desulfurization and reductive removal of the ketone gave access to **38d** (77%, two steps). Subsequently, a modified Barton–McCombie deoxygenation using chlorothionoformate **36e** was conducted and anisole demethylation (BBr_3_) resulted in the formation of 5‐*ep*i‐aureol (**39**). In order to obtain the meroterpenoid natural product **40**, it was necessary to assess the substitution pattern of the arene in five additional steps.

**Scheme 14:**
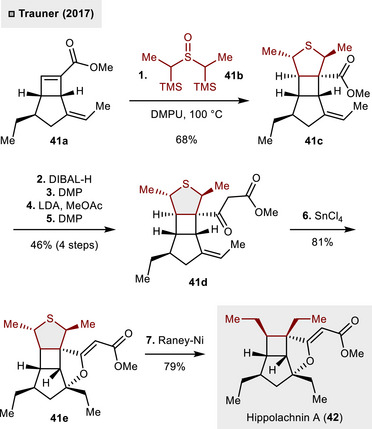
Total synthesis of hippolachnin A (**42**) making use of a THT intermediate for vicinal *cis*‐diethylation.

At the same time, a similar strategy has been applied by Trauner and coworkers in the total synthesis of hippolachnin A (**42**) as shown in Scheme 14.^[^ ^45^ ^]^ Herein, cyclobutene **31a** underwent the desired (3 + 2)‐cycloaddition with 1,3‐dipole precursor **41b** to install vicinal *cis*‐ethyl groups. Noteworthy, thermal reaction conditions (100 °C) were sufficient to effect this transformation and tricycle **41c** could be obtained in a satisfactory yield of 68%. The β‐ketoester **41d** was then synthesized in four steps through redox manipulation and aldol reaction with MeOAc. The synthesis was then completed via Lewis acid‐catalyzed cyclization employing SnCl_4_ to form the missing tetrahydrofuran motif (**41e**, 81%) and final desulfurization with Raney nickel, revealing the natural product in 79% yield.

**Scheme 15:**
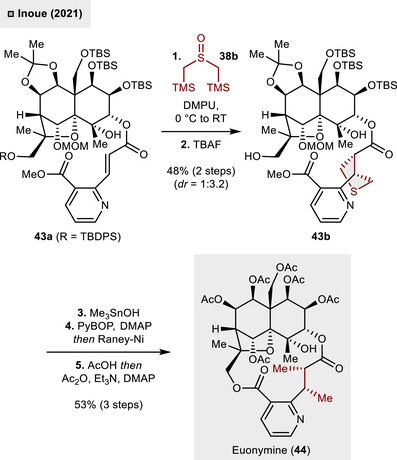
Late‐stage introduction of two methyl groups Through thiocarbonyl ylide (3 + 2)‐cycloaddition–desulfurization.

In 2021, thiocarbonyl ylide chemistry was employed by Inoue and coworkers in the total synthesis of the agarofuran natural product euonymine (**44**, Scheme 15).^[^ ^46^ ^]^ The highly oxygenated skeleton features two vicinal, *cis*‐oriented methyl groups. These alkyl residues were introduced through a 1,3‐dipolar cycloaddition of the electron‐poor olefin in **43a** and the fleeting thiocarbonyl ylide **C2** generated from **38b**. The reaction could be conducted at low temperatures (0 °C to RT) and delivered the envisioned THT derivative **43b** as the minor diastereomer (*dr*  =  1:3.2, 48%). This stands in sharp contrast to the previous synthetic applications of thiocarbonyl ylides which typically required thermal activation for strained or activated, electron‐deficient olefins and high‐pressure conditions for less‐activated dipolarophiles such as **38a**.^[^ ^44^ ^]^ However, the direct application of the (3 + 2)‐cycloaddition–desulfurization strategy to the macrolactone exclusively led to the undesired diastereomer. The synthesis was then finalized through a three‐step procedure (53% overall yield): Saponification with Me_3_SnOH set the stage for macrolactonization step (PyBOP, DMAP) and subsequent desulfurization revealed the two methyl groups. The final deprotection under acidic conditions (AcOH) followed by peracetylation culmulated in the total synthesis of euonymine (**44**).

**Scheme 16:**
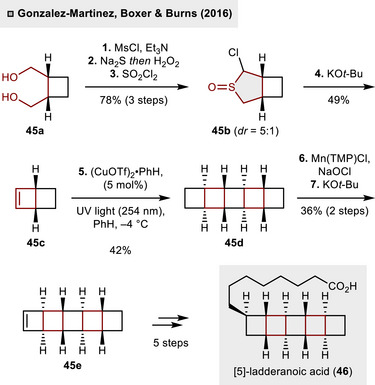
Chemical synthesis of [5]‐ladderanes via an atypical Ramberg–Bäcklund reaction.

In contrast to *cis*‐1,2‐dicarbofunctionalizations of olefins, THTs could also be employed as cyclobutene precursors, as demonstrated in the synthesis of [5]‐ladderanoic acid (**46**), depicted in Scheme 16.^[^ ^47^ ^]^ In 2016, Burns and colleagues identified a rather unusual Ramberg–Bäcklund reaction of α‐chloro sulfoxide **45b** as the key transformation to access bicyclohex[2.2.0]ene (**45c**). While the conventional Ramberg–Bäcklund reaction relies on the extrusion of SO_2_ from α‐halo sulfones,^[^ ^48^ ^]^ the use of **45b** – readily available in three steps from 1,4‐diol **45a** – was imperative to obtain superior yields of strained olefin **45a** (49%, 15 g scale). Noteworthy, this transformation has been rarely described in the literature and found to be highly depending on the base and equivalents utilized.^[^ ^49^ ^]^ In the present synthesis, it was uncovered that an excess of KO*t*‐Bu (three equivalents) was optimal and both diastereomers of **45b** (*dr* = 5:1) participated in olefin formation, rendering this inconsequential. The subsequent step involved the diastereoselective dimerization under UV‐irradiation (254 nm) at low temperature (–4 °C) with catalytic amounts of Cu(OTf)_2_ benzene complex (5 mol%). The obtained [5]‐ladderane **45d** was then subjected to a two‐step protocol toward cyclobutene formation through Mn‐catalyzed C(sp^3^)‐H chlorination and ensuing elimination employing KO*t*‐Bu (36% over two steps). To synthesize [5]‐ladderanoic acid (**46**), a total of seven additional steps were necessary involving the installation of the carboxylic acid‐pending side chain, thus completing the total synthesis. In addition, the divergent synthesis of [3]‐ladderanes was successfully demonstrated through the employment of benzoquinones for (2 + 2)‐cycloaddition with **45c**.

**Scheme 17:**
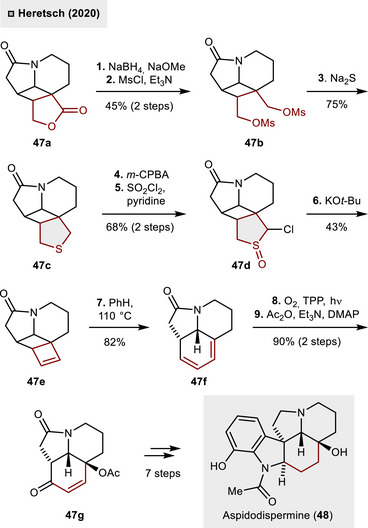
Total synthesis of aspidodispermine (**48**) through a pericyclic cascade reaction.

As illustrated in Scheme 17, Heretsch and coworkers applied a related strategy in their total synthesis of aspidodispermine (**48**).^[^ ^50^ ^]^ The 3,4‐fused DHT derivative **47c** was initially synthesized in three steps from lactone **47a** via reduction to the 1,4‐diol, twofold mesylation (**47b**) and ring‐closure upon exposure to Na_2_S. Subsequent oxidation to the corresponding sulfoxide (*m*‐CPBA) and α‐chlorination using SO_2_Cl_2_ resulted in a complex mixture of diastereomers (**47d**) in 68% yield over two steps. Treatment with KO*t*‐Bu then promoted the atypical Ramberg–Bäcklund reaction, thereby facilitating the synthesis of a highly strained cyclobutene motif **47e** through the extrusion of SO (43% yield). From there, thermal activation (110 °C) was employed to induce ring‐opening driven by ring‐strain release. This process resulted in the formation of cyclohexa‐1,3‐diene **47f** in 82% yield. With the 1,3‐diene in hand, the envisioned synthesis of α,β‐unsaturated ketone **43g** could be tackled. Exposure to singlet oxygen (^1^O_2_) – generated under white light irradiation and using tetraphenylporphyrin (TPP) as photosensitizer – led to smooth formation of an endoperoxide, which rapidly participated in Kornblum–DeLaMare rearrangement. The obtained tertiary alcohol was then acetylated yielding α,β‐unsaturated ketone **47g** in high yields (90% over two steps). From this key intermediate, the natural product aspidodispermine (**48**) could be obtained in additional seven steps involving the late‐stage installation of the indoline moiety.

## 5 Conclusion

Thiophene derivatives have been demonstrated to function as versatile C_4_‐building blocks in the construction of highly substituted ring systems through pericyclic transformations, including cycloadditions and electrocyclizations. The ability to generate highly complex scaffolds or quaternary stereocenters has rendered thiophene compounds valuable intermediates for the synthesis of natural products and bioactive molecules, that are of utmost importance for crop science and medicinal chemistry. Future research in this area will elucidate the capacity of thiophenes in the late‐stage diversification of drug candidates.

## Conflict of Interests

The authors declare no conflict of interest.

## Data Availability

The data that support the findings of this study are available from the corresponding author upon reasonable request.
